# *Bartonella* species in Cambodia, Ghana, Laos, and Peru: results from vector and sero-surveys

**DOI:** 10.1089/vbz.2021.0090

**Published:** 2023-01-01

**Authors:** Kristin Mullins, Enrique Canal, Pidor Ouch, Didot Prasetyo, Janice Tagoe, Naiki Attram, Clara Yeboah, Selassi Kumordjie, Anne Fox, Andrew G. Letizia, Audrey Rachlin, Hung Manh Nguyen, Matthew T. Robinson, Manivanh Vongsouvath, Viengmon Davong, Mayfong Mayxay, Mark P. Simons, Angela Caranci, Paul N. Newton, Allen L. Richards, Christina M. Farris

**Affiliations:** 1 University of Maryland School of Medicine, Baltimore, Maryland USA; 2 Naval Medical Research Unit-6, Lima Peru; 3 Naval Medical Research Unit-2, Phnom Penh Cambodia; 4 Naval Medical Research Unit-3 Ghana Detachment, Accra Ghana; 5 Lao-Oxford-Mahosot Hospital-Wellcome Trust Research Unit, Microbiology Laboratory, Mahosot Hospital, Vientiane Laos; 6 Center for Tropical Medicine & Global Health, Nuffield Department of Medicine, Oxford University, Oxford, United Kingdom; 7 Microbiology Laboratory, Mahosot Hospital, Qua Fa Ngum, Vientiane, Lao PDR; 8 Institute of Research and Education Development, University of Health Sciences, Ministry of Health, Vientiane, Lao PDR; 9 Naval Medical Research Center, Silver Spring, Maryland USA; 10 Uniformed Services University, Bethesda, Maryland USA; 11 Northwest Mosquito and Vector Control District, Corona, CA

## Abstract

*Bartonella* species are fastidious Gram negative vector-borne bacteria with a wide range of mammalian reservoirs. While it is understood that some species *Bartonella* are human pathogens, the extent of human exposure to *Bartonella* species (both pathogenic and non-pathogenic) has yet to be fully understood. To this end, residual sera from participants enrolled in undifferentiated fever studies in Cambodia, Ghana, Laos, and Peru were screened for the presence of IgG antibodies against *B. quintana* and *B. henselae*, using the FOCUS diagnostics Dual Spot- *Bartonella* IgG Immunofluorescence assay. Forty-eight patients with suspected or confirmed *B. bacilliformis* exposure or infection in Peru, were screened to assess cross-reactivity of the FOCUS assay for IgG against other *Bartonella species*. Ten of 13 patients with confirmed *B. bacilliformis* infection were *Bartonella*-specific IgG positive and overall, 36/48 of the samples were positive. Additionally, 79/206, 44/200, 101/180, and 57/100 of samples from Peru, Laos, Cambodia, and Ghana, respectively were *Bartonella*-specific IgG positive. Further, ectoparasites pools from Cambodia, Laos, and Peru were tested using quantitative real-time PCR (qPCR) for the presence of *Bartonella* DNA. Of the sand-fly pools collected in Peru, 0/196 were qPCR positive; 15/140 flea pools collected in Cambodia were qPCR positive; while 0/105 ticks, 0/22 fleas, and 0/3 louse pools collected in Laos tested positive for *Bartonella* DNA. Evidence of *Bartonella* in fleas from Cambodia supports the possibility that humans are exposed to *Bartonella* through this traditional vector. However, *Bartonella* species were not found in fleas, ticks, or lice from Laos, or sandflies from Peru. This could account for the lower positive serology among the population in Laos and the strictly localized nature of *B. bacillformis* infections in Peru. Human exposure to *Bartonella* species and *Bartonella* as a human pathogen warrants further investigation.

## Introduction

*Bartonella* species are fastidious, Gram negative, vector-borne bacteria. Classically, three *Bartonella* species have been identified as causing human bartonellosis, *B. bacilliformis*, *B. quintana*, and *B. henselae*, however a growing number of *Bartonella* species are being implicated in human disease. The genus grew from just one member with the discovery of *B. bacilliformis* in 1909, to five members in 1993, when the *Bartonella* and *Rochalimaea* genera were united(R L Regnery, Anderson, et al. 1992; Noguchi and Battistini 1926; Brenner et al. 1993; Daly et al. 1993; Russell L Regnery, Olson, et al. 1992). Since then, the number of known *Bartonella* species has grown to more than fifty-five current and *Candidatus* members(Breitschwerdt 2017; K. E. Mullins et al. 2015; Mangombi et al. 2020; Gutiérrez et al. 2020; Medkour et al. 2019). *Bartonella* species have been isolated from a wide variety of invertebrates, including fleas, ticks, body lice, sheep keds, and even spiders, and hosts including humans, cats, dogs, rats, cattle, foxes, and bats(Breitschwerdt 2017). Importantly, at least 17 species have now been implicated in human disease(Breitschwerdt 2017; K. E. Mullins et al. 2015).

Disease presentation varies among *Bartonella* species. *B. bacilliformis* infection, thought to be transmitted by sandflies, is limited to the Andes mountain region of South America at elevations of 500-3,600 meters above sea level and causes Carrion’s disease, which includes an acute hemolytic syndrome (Oroya fever) as well as a chronic condition (verruga peruana)(Jacomo, Kelly, and Raoult 2002; C. Gomes and Ruiz 2018; Clemente et al. 2012). *B. quintana*, transmitted by human body lice, and *B. henselae*, transmitted by fleas have worldwide distribution and are classically known to cause trench fever, known for its hallmark symptom, a relapsing fever, and the self-limited infection cat scratch disease, which presents as a fever with swollen lymph nodes, respectively(Jacomo, Kelly, and Raoult 2002; Karem, Paddock, and Regnery 2000). More recently, *Bartonella* species have been implicated in a wide range of disease manifestations from asymptomatic infection to mild fever and maculopapular rash to severe disease such as endocarditis, hallucinations, and even death(Breitschwerdt 2017; Cheslock and Embers 2019).

Due to the fastidious nature and lack of awareness of *Bartonella* species as human pathogens, the true extent of human exposure and disease associated with this genus is not fully understood(Cheslock and Embers 2019; Jacomo, Kelly, and Raoult 2002). Worldwide distribution and the increasing number of *Bartonella* species implicated in human disease suggests that *Bartonella* infections likely play a larger role in human disease than currently recognized(Cheslock and Embers 2019). To gain a better understanding of human exposure or the potential for human exposure to *Bartonella* species sero-surveys for IgG antibodies against *Bartonella* species and arthropod vector-surveys for molecular detection of *Bartonella* species in fleas, ticks, and lice were conducted in Cambodia, Ghana, Laos, and Peru.

## Materials and Methods

### Serosurveys

Human sera from undifferentiated fever studies conducted in Cambodia, Ghana, Laos, and Peru were tested for the presence of IgG antibodies targeting *Bartonella* species to elucidate previous or recent exposure to *Bartonella*. These serosurveys were conducted using the FOCUS Diagnostics *Bartonella* IFA IgG test (Cypress, California USA) per manufacturer’s instructions. Positive and negative controls were included on each slide. Samples were screened at a 1:64 dilution and a positive result was recorded if a 1+ apple green fluorescence was observed for the *Bartonella quintana* and/or *Bartonella henselae* antigens. Slides were reviewed by at least two researchers and samples were considered positive only if both researchers agreed.

The FOCUS Diagnostics IFA IgG test uses Vero cells infected with *B. henselae* and *B. quintana*, however, those individuals infected with *B. quintana* and *B. henselae* show high IgG cross-reactivity on testing and there is well documented, albeit variable, cross-reactivity in patients infected with numerous other *Bartonella* species, aiding in the diagnosis of Bartonellosis in these patients. (Chamberlin et al. 2000; Maurin, Rolain, and Raoult 2002; Sander et al. 1998; Oteo et al. 2017; Roux et al. 2000; Iralu et al. 2006). Cross-reactivity, is likely due to the use of whole cell *Bartonella* antigens from *B. quintana* and *B. henselae*, antigens that would be present in both pathogenic and non-pathogenic *Bartonella* species. Due to this, positivity on the FOCUS IFA IgG test is could indicate exposure to both pathogenic and/or non-pathogenic *Bartonella*.

#### Cambodia

One hundred and eighty serum samples were collected by the Naval Medical Research Unit 2 detachment Phnom Penh between January 2013 and December of 2014 from febrile patients in Rattanakiri, Kratie, Stung Treng, Svay Rieng, Kampong Speu, and Kandal provinces after informed consent(Inghammar et al. 2018). Samples were storred at -70°C until use.

#### Ghana

One hundred serum samples were collected by the Naval Medical Research Unit 3 Ghana detachment located in Accra between 2008-2009 from patients in Ashanti, Easter, and Greater Accra Regions of Ghana after written informed consent (Tagoe, et al, unpublished). Samples were storred at - 70^0^C until use.

#### Laos

One-hundred and ninety-nine serum samples were collected by the Microbiology Laboratory/Lao-Oxford-Mahosot Hospital-Wellcome Trust Research Unit in July and August of 2014 after written consent from inpatients undergoing investigation for the etiology of fever at Mahosot Hospital in Vientiane Capital of Laos(Phetsouvanh et al. 2006). Samples were storred at -70^0^C until use.

#### Peru

Serum samples from 48 patients were collected during a 2003 outbreak of *B. bacilliformis* in Huancambamba Province. Samples were collected from patients with suspected *B. bacilliforms* infections. Thirteen of the suspected cases were confirmed by PCR, culture, and/or identification of B. bacilliforms in blood smears. Samples were screened to assess the extent of the exposure to *B. bacilliformis* and to assess the ability of the assay to detect anti-*B. bacilliformis* IgG antibodies. Further, 206 serum samples collected between 2008 and 2015 from individuals with undifferentiated fever in the Amazonas, Junín, Cusco, and Loreto Regions of Peru were screened for the presence of IgG antibodies to *Bartonella* species(Santiago et al. 2015). Sample intergrity was assessed by were storred at -70^0^C until use.

### Vector-Surveys

DNA extracted from fleas, lice, sandflies, and from previous vector studies was used for the detection of *Bartonella* species.

In Cambodia, 140 flea pools (2 to 25 fleas/pool) were collected from November of 2015 through January of 2016 using combing/brushing technique from dogs, cats, rats and mice found in Rantanakiri and Stung Treng Provinces. Of those 14 pools of *Ctenocephalides felis* and *Pulex irritans* were collected from cats, 119 pools of *C. felis*, *Echidnophaga gallinacea*, *P. irritans* were collected from dogs, and 7 pools of *Xenopsylla cheopis* were collected from mice and rats. Fleas were preserved in 70% ethanol, stored in cooler box, and transferred to laboratory. Fleas were morphologically identified using standard taxonomic keys and pooled by species and location (Menier and Beaucornu, 1930) . Fleas were briefly washed with distilled water and the DNA were extracted using DNeasy blood and tissue kit (Qiagen, Germany) following manufacturer’s instruction. Sample intergrity was assesed by Nano-Drop spectrophotometer (Thermo Scientific, Waltham, MA, USA) and were storred at -70^0^C until testing.

In Laos, fleas, ticks and lice were collected in March of 2018 from dogs in Vientiane City and pooled based on species and host, resulting in 22 flea pools, 106 tick pools, and 3 louse pools. Flea and louse pools contained 2-20 individual *C. felis* fleas or *Heterodoxus spiniger* lice, while 210 *Rhipacephalus sanguineus* ticks were pooled. DNA was extracted and stored as described in Nguyen et al, 2020. (Nguyen et al. 2020)

In Peru, sandflies were collected in the Ancash region in 1999 and the Cajamarca region in 2012-2013 resulting in 99 and 100 pools of 2-10 sandflies/pool based on morphological detection, respectively (Romero 2004; Zorrilla et al. 2021). Sandflies were stored in 70% ethanol at -70^0^C until DNA was extracted. DNA were extracted using DNeasy blood and tissue kit (Qiagen, Germany) following a protocol modified from the manufacturer’s instructions (Depaquit et al. 2005). DNA was stored at -70^0^C and sample intergity was assessed by Nano-Drop spectrophotometer.

*Bartonella* species were detected using a *Bartonella* genus-specific qPCR assay, BartA, as described in Flores Mendoza et al, 2021 which targets the *ftsZ* gene(Flores-Mendoza et al. 2021). In brief, each reaction contained 0.7 μM of the forward and reverse primers and 0.4 μM of the probe. Three microliters of template DNA was added to each reaction. The cycler parameters included an initial incubation at 50°C for 2 minutes followed by an initial denaturation at 95°C for 2 minutes, then 45 cycles of denaturation at 95 °C for 15 seconds and annealing/elongation at 60 °C for 30 seconds. *Bartonalla ancashensis* genomic DNA was used as the positive control.

### Statistical analysis

Data managment and statistical analysis was performed in Microsoft (Redmond, WA) Excel. Minimum infection rate (MIR) was caculated: ([number of positive pools / total specimens tested] x 1000).

### Ethical approvals

This study was approved by the Naval Medical Research Center Institutional Review Board in compliance with all applicable federal regulations governing the protection of human studies (NAMRU2.2005.004, NAMRU3.2016.0006, NMRCD.2010.0010, NMRCD.2001.0006, NMRCD2010.0010, NMRC-IRB NAMRU3.2014.0003).

For Ghana, the protocol was also approved by the Noguchi Memorial Institute for Medical Research (NMIMR) IRB

For Laos, ethical approval was granted by the National Ethics Committee for Health Research (Lao PDR) and the Oxford Tropical Research Ethics Committee (UK).

## Results

### Serosurveys

Seropositivity rates among febrile patients ranged from 22% in Laos to 57% in Ghana (Figure 1; Table 1). In Laos, IgG antibodies against *Bartonella* species were detected in febrile patients admitted to Mahosot Hospital with homes in Borikhamxay (3/11), Champassack (1/1), Luangnamtha (2/3), Luangphrabang (1/6), Oudomxay (1/2), Vientiane Capital (25/135), Vientiane Province (9/32), Xaiyabuly (1/2), and Xiengkhuang (1/4) provinces, but not in Attapeu, Huaphan, and Sekong Provinces where only one sample was tested from each. The overall sero-positive rate in Peru was slightly higher with 38% of samples collected from patients with undifferentiated fevers from the Amazonas (2/3), Junin (29/69), Cusco (24/66), and Loreto (24/68) regions of Peru positive for IgG antibodies against *Bartonella* species. Of the 180 serum samples from febrile patients tested in Cambodia 56% (101) were positive including samples from Kampong Speu (26/42), Kandal (12/22), Kratie (11/15), Rattankiri (3/13), Stung Treng (3/7), and Svay Rieng (46/81) provinces. While in Ghana, 57% of the samples were positive for IgG antibodies wherein (2/3) and (55/96) of samples from febrile patients from the Eastern and Greater Accra region were positive, respectively, while the single sample from the Ashanti region was negative. (Figure 1; Table 1)

Interestingly, overall, there was no statistical signifiance between individuals sex and presence or absense of antibodies to Bartonella species in Cambodia, Ghana, Laos, and Peru. Additonally, there was no association between age and presence or absence of antibodies in Cambodia, Laos or Peru, however in Ghana, individuals positive for antibodies against *Bartonella* species were older, 37.8 mean age compared to 30.5 (p=0.037) for those without antibodies aganist *Bartonella* species. (Table 2)

Separately, serum samples from patients with confirmed or suspected *B. bacilliformis* infection from the 2003 outbreak in Piura, Peru were also tested to confirm cross-reactivity of the FOCUS assay with antibodies to *B. bacilliformis*. Ten of the 13 (77%) serum samples from patients with confirmed *B. bacilliformis* infection were positive for IgG antibodies to *Bartonella* species. Interestingly, 75% (36/48) of individuals with suspected *B. bacilliformis* infection were positive for IgG antibodies to *Bartonella* species.

### Vector-Surveys

The minimum infection rate of the flea pools from dogs, mice and rats found in the Ratanakiri and Strung Treng provinces of Cambodia was found to be 13.37% (0-39). Surprisingly, no flea pools collected from cats in the same provinces were positive. Flea species positive for *Bartonella* species DNA included *C. felis*, *P. irritans*, and *X. cheopis*. Pools from ticks (0/106), fleas (0/22), and lice (0/3) collected from dogs in Vientiane Capital of Laos were all negative for *Bartonella* species DNA. Further, none of the 196 pools of sandflies collected in Ancash and Cajamarca regions of Peru were positive for *Bartonella* species DNA. (Figure 1; Table 3)

## Discussion and Conclusions

*Bartonella* species are increasingly common and recent studies have highlighted the widespread nature of *Bartonella* species (both human pathogens and non-human pathogens) infecting vectors such as fleas, ticks, lice and small mammal reservoir hosts worldwide (Cheslock and Embers 2019). The molecular detection of *Bartonella* species in flea pools collected from dogs, mice, and rats from Ratanakiri and Stung Treng provinces of Cambodia in this study is to our knowledge the first report of *Bartonella* species in fleas from Cambodia. However, a few studies of rodents and small mammals from Cambodia have found molecular evidence of *Bartonella* infection ranging from 7-10% (Duong et al. 2013; Jiyipong et al. 2015). Our data from fleas coupled with the data supporting *Bartonella* species present in small mammals and rodents provides evidence of the widespread distribution of *Bartonella* species (pathogenic and/or non-pathogenic to humans) in Cambodia.

However, surprisingly, flea and louse pools collected from the dogs in Vientiane Capital, Laos were negative for *Bartonella* species DNA. Previous studies in Laos found evidence of *Bartonella* species in fleas (including fleas collected from dogs) with positivity rates ranging from 3.3- 33%, but these studies were outside of Vientiane Capital and there is evidence that levels of infection in rodents are significantly lower in human settlements(Varagnol et al. 2009; Jiyipong et al. 2015; Kernif et al. 2012; Calvani et al. 2020). Further, *Bartonella* species DNA was not found in ticks from Vientiane Capital, which is consistent with a previous study in which *Bartonella* species DNA was not detected in ticks collected from dogs and cats in Northern Laos(Kernif et al. 2012). While studies in the USA, Italy, Korea, and Austria have found evidence of *Bartonella* species in ticks ranging from 0.1 to 34.5%, ticks are not implicated as a major vector for human transmission of *Bartonella* species (Telford III and Wormser 2010; Persichetti et al. 2016; Satta et al. 2011; Kim et al. 2005; Adelson et al. 2004; Chang et al. 2001; Müller et al. 2016). An unfortunate and major limitation of our molecular surveys was the inability to provide sequence confirmation or speciation of *Bartonella* in these vectors and determine if the *Bartonella* species present in the animal reservoir or vector are pathogenic to humans. Nevertheless, this molecular evidence of *Bartonella* species in Southeast Asia supports previous reports and provides additional context for the results of the accompanying serosurveys and overall human exposure to *Bartonella* in Asia and worldwide.

While there are many studies of vectors and reservoir hosts, fewer studies investigate the prevalence of human exposure to (past or recent), or infection with, *Bartonella* species. Human *Bartonella* infections are described in case reports, most commonly endocarditis and cat-scratch disease, analysis of hospital records and insurance data, and from serosurveys with positivity ranges from 1% for healthy individuals in Sweden to 83% for sanitary workers in Spain(Daly et al. 1993; K. E. Mullins et al. 2015; Oteo et al. 2017; Müller et al. 2016; Im et al. 2018; Kwon et al. 2017; Portillo et al. 2020; Chomel et al. 2006; Bhengsri et al. 2011; MARUYAMA et al. 2000; Comer et al. 1996; Sun et al. 2010; Edouard et al. 2015; Fenollar, Sire, and Raoult 2005; FOURNIER et al. 2001; Kristin E. Mullins et al. 2013; Nawrocki et al. 2020; Alonso et al. 2020; Sandoval et al. 2020; Allizond et al. 2019). In this study, recent or previous *Bartonella*exposure was evaluated in febrile patients in Cambodia, Ghana, Laos, and Peru. To our knowledge these studies represent the first human serosurveys in Cambodia and Laos, and only the second study in Ghana, where the first focused on bat-associated *Bartonella* (Mannerings et al. 2016). In this study the overall seroprevelance in the countries surveyed was consistent with previous reports (which vary widely) and ranged from 22-57%, although results from our serosurvey could overestimate prevalence of antibodies in the general (or healthy) populations due to the possibility that some patients with undifferentiated fevers were actively infected with *Bartonella*. Interestingly, Laos had the lowest seroprevelance, which could be accounted for with the lack of molecular detection of *Bartonella* species in fleas in Vientiane Capital, and possibly why the first reports of *B. henselae* endocarditis were only reported in 2014 (Rattanavong et al. 2014). Higher seroprevelance was seen in Cambodia and Ghana at 56% and 57%, respectively. Differences in seroprevelance likely correspond with lifestyle, occupation, and vector positivity rates, and contact with vectors and reservoir hosts, among other risk factors. This study was not able to elucidate possible risk factors for *Bartonella* exposure due to limited data on the individuals for which serum was available. However, this additional evidence of human exposure to *Bartonella* from places with limited previous research provides support that larger studies to determine the impact of *Bartonella* on human heath are needed especially in Cambodia, Laos, and Ghana. Further, this evidence argues for more aggressive evaluation of *Bartonella* species as a potential causes of the undifferentiated febrile illness in these locations especially upon report of an exposure to a known vector.

Finally, exposure to *Bartonella* species in Peru is unique as the *Bartonella* species of major importance, *B. bacilliformis*, which causes both acute and chronic infections, is found only in the Andes Mountain range, and thought to be vectored by sandflies(C. Gomes and Ruiz 2018; Clemente et al. 2012). Since the FOCUS Diagnostics IFA uses *B. quintana* and *B. henselae* antigens, cross-reactivity with anti- *B. bacilliformis* IgG was assessed using serum samples from patients with known *B. bacilliformis* infection. Antibodies were detected in 77% (10/13) of these individuals at the 1:64 screening dilution. Not only does this indicate high infection rates, but that the FOCUS test has high cross-reactivity to Bartonella species indicating positivity on a FOCUS test would not be limited to *B. quintana* or *B. henselae* infections. Examination of the serologic evidence from a larger subset of individuals, those suspected of *B. bacilliformis* infection during the 2003 outbreak, shows widespread exposure to *Bartonella* (likely *B. bacilliformis*), with 75% of individuals seropositive, while *B. bacilliformis* was only identified by smear, PCR or culture for 27% of individuals. This indicates that smear, PCR, and culture may currently miss a large portion of infected individuals, or that many of these individuals had prior exposure, but not active infection. Given the cross-reactivity observed among known *Bartonella* species in serological assays, including the FOCUS IFA used in this study, the study participants may have been exposed to *Bartonella* species that have yet to identified or characterized, or even those that do not cause illness in humans.

This highlights the need for more in depth investigations into the *Bartonella* species in humans, animals, and vectors in countries such as those included in this study, but also in countries and regions where knowledge of these agents is lacking.

Further, *Bartonella* species DNA was not identified in any sandfly pools from the endemic areas of Ancash and Cajamarca. Interestingly, all previous studies have found low levels of *B. bacilliformis* infection of sandflies ranging from 0.5-2.8 %(Ulloa et al. 2018; Ellis et al. 1999). While *L. verrucarum* is considered a known vector of *B. Bacilliformis*, other sandfly species have been implicated as possible vectors including *L. maranosesis* and *L. robusta*, both of which were included in this study, however the mechanisms by which *B. bacilliformis* is transmitted by sandflies is still lacking (Ulloa et al. 2018; Ellis et al. 1999; Garcia-Quintanilla et al. 2019; Clemente et al. 2012). The absence of molecular detection of *Bartonella* species in sandflies from endemic areas provides more insight into the epidemic nature of *B. bacilliformis*, and possibly *B. ancashensis*, for which a vector has yet to be identified (Kristin E Mullins et al. 2017; K. E. Mullins et al. 2015). Outbreaks of *B. bacilliformis* could be driven by asymptomatic carriers and immediate transmission between persons via sandflies. Studies have shown that outbreaks occur within families and members of a household are 2.6 times more likely to contract *B. bacilliformis* than neighbors (Cláudia Gomes et al. 2016). Additionally, asymptomatic carriage of *B. bacilliformis* is known to occur, with up to 40% of individuals in a post-exposure setting positive by qPCR (Chamberlin et al. 2002; Cláudia Gomes et al. 2016). The seroprevelance rate of 38% for *B. bacilliformis* in individuals with undifferentiated fever endemic areas is consistent with previous studies of post-exposure or endemic asymptomatic populations which found seropositivity rates between 19-67% (Cláudia Gomes et al. 2016; Chamberlin et al. 2002; Knobloch et al. 1985; Chamberlin et al. 2000). Our data likely includes both exposure to *B. bacilliformis*, as seen during the outbreak, as well as other *Bartonella* species including those which are not known to be human pathogens. Nevertheless, this data combined with previous reports indicates exposure to *Bartonella* in Peru is of continuing importance.

This study combined with previous data indicates that human exposure to *Bartonella* species is extensive and that *Bartonella* species may be the cause of undifferentiated fever in a subset of the individuals from these studies. These data taken along with previous work indicate that *Bartonella* species are likely an under-represented as cause of human disease around the world.

## Figures and Tables

**Table 1:** Serologic Surveys for Anti-*Bartonella* Antibodies

Country	Dates of Collection	Province/Region	# of Samples	# of Positive Samples (%)
		Amazonas	3	2 (67%)
		Junín	69	29 (42%)
	2008-2015	Cusco	66	24 (36%)
		Loreto	68	24 (35%)
Peru		**All**	**206**	**79 (38%)** (53% both, 39% BH, 8% BQ)
	2003 outbreak of *B. bacilliformis*	Piura (Huancabamba)	35	26 (74%)
	2003 confirmed cases of *B. bacilliformis*		13	10 (77%)
	**All**		**48**	**36 (75%)**
Laos	2014	**Vientiane Capital**	**199**	**44 (22%)** (18% both, 79% BH, 3% BQ)
		Kampong Speu	42	26 (62%)
		Kandal	22	12 (55%)
		Kratie	15	11 (73%)
Cambodia	2013-2015	Rattanakiri	13	3 (23%)
		Stung Treng	7	3 (43%)
		Svay Rieng	81	46 (57%)
		**All**	**180**	**101 (56%)** (58% Both, 42% BH only)
		Ashanti	1	0 (0%)
Ghana	2008-2009	Eastern	3	2 (67%)
		Greater Accra	96	55 (57%)
		**All**	**100**	**57 (57%)**

Both -Reactive to *Bartonella henselae* and *quintana* antigens, BH- reactive to *Bartonella henelase* antigens only, BQ- reactive to *Bartonella quintana* antigens only

**Table 2:** Age and Sex distribution for individuals tested for Anti-*Bartonella* IgG

Country	Demographic	n (positive)	Overall	Positive	Negative	p-Value
	Age (years), mean (SD)	180(101)	17.4(14.4)	18.5 (15)	16(13.5)	0.245
Cambodia	Male Sex n (%)		90 (50)	48 (48)	42 (53)	0.453
	Age (years), mean (SD)	100(58)	34.6(15.3)	37.8 (15.3)	30.5(14.4)	0.037
Ghana	Male Sex n (%)		50(50.5)	26(46.4)	24(55.8)	0.354
	Age (years), mean (SD)	199(44)	35.3(25)	31.9(22.9)	36.2(25.5)	0.32
Laos	Male Sex n (%)		118(59.3)	28(63.6)	90(57.7)	0.117
	Age (years), mean (SD)	206(79)	28 (14.9)	30.24 (14.9)	30(15)	0.91
Peru (2008-2015)	Male Sex n (%)		90 (43.7)	28(35.4)	62(48.8)	0.083

**Table 3:** Ectoparasite Surveys: Molecular Detection of *Bartonella* species

Country	Dates of Collection	Province/Region	Host	# per Pool	Ectoparasite	Ectoparasite Species	# of Pools	# Positive Samples (%)
Peru	1999	Ancash	N/A	1-10	Sandlfies	Sandfly spp	99	0 (0%)
	2012-2013	Cajamarca				*L. castanea*	19	0 (0%)
						*L. maranosesis*	59	0 (0%)
						*L. robusta*	19	0 (0%)
						*L. verrucarum*	3	0 (0%)
			**All**				**199**	**0 (0%)**

Cambodia	2015-2016	Ratanakiri	Cats		Fleas	*C. felis*	11	0 (0%)
						*P. irritans*	1	0 (0%)
			Dogs			*C. felis*	63	1 (1.6%)
						*E. gallinacea*	4	0(0%)
						*P. irritans*	29	9 (30%)
			Mice			*X. cheopis*	2	0 (0%)
		Stung Treng	Cats			*C. felis*	2	0 (0%)
			Dogs			*C. felis*	23	1 (4.3%)
			Mice			*X. cheopis*	3	2 (67%)
			Rats			*X. cheopis*	2	2 (100%)
			**All**				**140**	**15 (11%)**

Laos	2014	Vientiane Capital	dogs	1-10	Ticks	*S. sanguineus*	106	0 (0%)
				2-20	Fleas	*C. felis*	22	0 (0%)
					Lice	*H. spiniger*	3	0 (0%)
			**All**				**136**	**0 (0%)**
